# Effect of Triphala on growth, immunity, related gene expression and intestinal morphometry of yellow perch (*Perca flavescens*)

**DOI:** 10.1371/journal.pone.0315305

**Published:** 2025-03-04

**Authors:** Hiam Elabd, Han-Ping Wang, Rafidah Othman, Hong Yao

**Affiliations:** Ohio Center for Aquaculture Research and Development, The Ohio State University South Centers, Piketon, Ohio, United States of America; Mansoura University, EGYPT

## Abstract

The current study evaluated the effects of dietary supplementation of Triphala (TR) on yellow perch (*Perca flavescens*) growth performance, immune response, related gene expression, and intestinal histological structure. The experimental design included four groups: one control group (0% TR/ kg diet) and three TR-supplemented groups with 2, 4, and 6%/kg diet for four weeks and each group was allocated in triplicates with 30 fish each. Sampling included three fish from each replicate for evaluating immune response and gene expression. Findings showed that Triphala markedly improved growth performance, Immunoglobulin M (IgM) levels, lysozyme activity, and Nitric Oxide (NO) activity with the most significant (p < 0.05) results for 6% TR/kg diet group. The TR groups also showed significantly decreased glucose and cortisol concentrations with the lowest values for the 6% TR/kg diet group. Moreover, TR-incorporated groups revealed significantly upregulated expression (p < 0.05) of growth [Insulin-Like Growth Factor-1 (IGF-1)] and immune [Alpha 2 Macroglobulin (A2M), Serum Amyloid A (SAA) and Complement Component C3 (CCC3)] genes in incorporated groups, specially the 6% TR group. Moreover, the intestinal morphometric histological analysis revealed that villus length was increased in a dose-dependent manner, coping with other enhanced parameters. Current results endorse the positive effects of Triphala incorporation on yellow perch farming as a safe alternative option to enhance growth performance, immune response, related gene expression, and intestinal histology.

## 1. Introduction

Aquaculture faces several challenges that can compromise fish health status including infections and management of stressful conditions [1–5]. Yellow perch (*Perca flavescens*) is one of the most common fish species in the eastern part of North America [6]. Currently, attention is being paid toward the use of natural phytogenic compounds (i.e., natural plant herbal compounds) as efficient alternatives to antibiotics and chemical therapies that are proven to promote growth, antioxidative, and immune responses, resistance [7–9]; and manipulate gut histology [10–14]. The intestinal histology could be indicative to the effect of natural dietary supplementation on fish health status through assessing the immune response, growth performance, and biochemical profiles [9,12,13,15].

In the current study we focused on addressing the effect of dietary Triphala incorporation in fish diets, which is a natural polyherbal formula prepared by mixing three dried powder herbs at rate of 1:1:1 (namely, *Emblica officinalis, Terminalia chebula* and *Terminalia bellerica*), on growth, physiology and immune responses in yellow perch. Triphala possess growth and immune-promoting effect, detoxification, and diseases resistance in fish [16,17]. This growth-promoting effect could be due to the effect of its content of active herbal ingredients, specially rich amino acids, fatty acids, carbohydrates [18,19].

In terms of evaluating those responses, IGF-I is a key indicator for growth performance and regulation [7,20], while IgMtetramer is the predominant immunoglobulin in teleost and contains eight antigenic combining sites [21]. Alpha 2-macroglobulins (A2M) are proteinase inhibitors that are present in the plasma of vertebrates and invertebrates. They also function as a binding protein to numerous growth factors, cytokines and hormones [12,21]. Serum amyloid A (SAA) plays an important role in inflammatory cellular response as a member of acute phase proteins that recruits chemotactic cells in the inflammatory sites. Complement Component C3 (CCC3) is also a complement protein that helps in generation of chemotactic factors, immune responses and complexes [20,22,23].

As far as we know, no previous studies addressed the effect of Triphala on yellow perch or on its intestinal morphometry. Thus, the current study was designed to evaluate the effect of Triphala dietary supplementation in yellow perch diets on growth performance, immune response, gene expression, and intestinal histological analysis.

## 2. Materials and methods

The current experiment was performed according to the guidelines of The Ohio State University for laboratory animal care and use. All procedures assured that all applied management practices were done while avoiding stress exposure and reducing whenever possible its level to the minimal during the experiment. All personnel were adequately trained on fish handling and different husbandry procedures.

### 2.1. Diets

Triphala^®^ was obtained from Banyan Botanicals (New Mexico, USA). AquaMax grower 400^®^ (Purina Animal Nutrition, MO, USA; Crude Protein Minimum 45.0%, Crude Fat Minimum 16.0%, Crude Fiber Maximum 3.0%, Calcium Minimum 2.2%, Phosphorous (P) Minimum 1.2%, Sodium (NA) Minimum 0.6%) was divided into four portions. The first was kept as a control without any additives. The remaining portions were mixed with 2, 4, and 6% Triphala/kg, respectively. 100 ml water was added/kg basal diet, mixed thoroughly and extruded (2 × 3 mm) using a XLS turbo force extruder 3000 (XLS International, NE, USA). After that, pellets were spread on trays and left for 24 h air drying, then dried pellets were stored in plastic Ziploc bags at −20°C.

### 2.2. Experimental fish and design

Yellow perch of average initial weight 18.1 ± 2.9 g and initial length 11.1 ± 0.7 cm were obtained from the hatchery of the Ohio Center for Aquaculture Research and Development at The Ohio State University South Centers, Ohio, USA and randomly distributed into four groups in 50 L fiberglass round tanks in triplicates (90 fish/group, 30 fish/replicate). Acclimation to the experiment conditions took two weeks and fish were fed to satiation two times daily (09.00 am and 16:00 pm) for four weeks. Throughout the experiment, water temperature was 20.83 ± 0.3°C and dissolved oxygen content was 7.56 ± 0.4 mg/L. Both parameters were measured twice daily and photoperiod was set to 12 h light/12 h darkness cycle. We confirm that the entire experimental flow and fish samples were in compliance with the experimental and animal care procedures that were approved by the Institutional Animal Care and Use Committee of the Ohio State University.

### 2.3. Sampling

At the end of the four weeks, fish anesthetization was performed using 250 ppm MS222 (Syndel Laboratories Ltd., British Columbia) and blood samples were collected (n = 9/group, 3 samples/replicate) from caudal vessels using a 1 ml syringe (Becton Dickinson, USA) containing EDTA into EDTA-coated tubes and kept on ice until 5 minutes centrifugation at 3,600 rpm and 4 °C to obtain plasma following our laboratory protocols (Elabd et al., 2020, 2017, 2016a, 2016b) for the evaluation of glucose, cortisol, IgM levels, lysozyme activity, and nitric oxide activities.

Liver samples were carefully isolated from same fish, washed with phosphate-buffered saline (PBS, pH 7.4), homogenized in 50 mM PBS (containing 1 Mm EDTA, pH 7.4) as mentioned previously by Elabd et al. (2020, 2017, 2016a, 2016b), centrifuged (15,000 × g, 10 minutes, 4ºC), and the supernatant was stored at −80 ºC until evaluating IgM and nitric oxide levels. Another same set of liver samples (n = 9/group, 3 samples/replicate) were collected in RNAlater (Ambion, USA) and stored at −80 °C for subsequent gene expression study.

### 2.4. Growth indices

At the stocking and after four weeks, specific growth rate (SGR), body mass gain (BMG), length gain rate (LGR), feed conversion ratio (FCR), and hepatosomatic index were measured as follows: (SGR % day^−1^) = [(ln final body mass (g)) - ln initial body mass (g))/number of experimental days] X 100, BMG % = 100 × [final body mass (g) - initial body mass (g)]/ initial body mass (g)], LGR % = 100 × [average terminal body length (cm) – average initial body length (cm)]/average initial body length (cm), FCR = *F*/(*Wf _ Wi*); where *F* is the given feed weight, *Wf* is the fish final weight, and *Wi* is the fish weight at stocking and HSI = [liver weight (g)/total body weight (g) × 100].

### 2.5. Immune-related parameters

IgM, lysozyme activity, nitric oxide, and myeloperoxidase activity levels were measured using a Microplate spectrophotometer (Epoch™, USA). IgM levels were measured at 450 nm using Fish IgM ELISA kit (MyBioSource Inc., USA). Lysozyme activity was measured kinetically by recording the decrease in A_450_ for 5 min using a Lysozyme detection kit (Sigma-Aldrich, USA). Nitric oxide was assayed at 540 nm using commercial kit (BioVision Inc., USA) according to the manufacturer’s protocol.

### 2.6. Glucose and cortisol assays

Plasma glucose and cortisol concentrations assays were performed in triplicates at 450 nm using (Epoch™ Spectrophotometer, USA). Glucose level was measured following the glucose assay kit’s protocol (Biovision, USA) as previously mentioned in our studies (Elabd et al., 2017, 2016a, 2016b) and cortisol according to (MyBioSource Inc., USA) manufacturer’s protocol.

### 2.7. Expression of IGF-1 and immune related genes

Total RNA was extracted from liver samples (n = 9/group, 3 fish/replicate) after four weeks feeding Triphala-incorporated diets using TRIzol (Invitrogen, USA) following the company’s protocol. Extracted RNA was DNase treated and concentration was checked using a Nano-Drop spectrophotometer (Thermo Scientific, USA) and purity by OD _260_/_280_ nm ratio that ranged 1.80: 2.00; and then reverse transcripted using a high-Capacity cDNA Reverse Transcription Kit (Invitrogen, USA), following Elabd et al. (2020, 2017a, 2016a, 2016b) according to the manufacturer’s protocol for 20 μL volume. cDNA yield was stored at −20°C. IGF-1, SAA, CCC3, A2M and β-actin genes primers (Table 1) were previously designed and tested (Elabd et al., 2017, 2016a) using NCBI’s Primer–BLAST. 20 μL volume Real-time PCR analysis was performed in triplicates in 7500 Real-Time PCR System (Applied Biosystems®, USA) using SYBR select Master Mix (Applied Biosystems, USA).

**Table 1:** Primers sequences for housekeeping gene and genes of interest evaluated in the gene expression analysis of perch*.*

Gene of interest	Forward primer sequence (5’-3’)	Reverse primer sequence (5’-3’)
β-actin	GCCTCTCTGTCCACCTTCCA	GGGCCGGACTCATCGTACT
IGF-1	AGTACCGCAGGGCACAAAGT	CTGGCTGCTGTGCTGTCCTA
SAA	ACCATGCTCGTTTGCCTTCT	TGTGGCGAGCATACAGTGAT
CCC3	GCACAGGAGAAGCAACAGTG	AGGAGCTGCACTGACAAGTTA
(α 2-M)	TACAGGAGCACCAAGTGCAG	GACTGACCACACGCTCTTCA

Relative expression level of the test sample’s target gene to that of the calibrator was calculated using the “ΔΔCt” method mentioned by [9,12,20,21,24].

### 2.8. Intestinal villi absorptive capacity morphometrical assessment

Intestinal samples were submitted for histological analysis (nine samples/group, three fish/ replicate). Samples were collected in Prefer Fixative (Fisher scientific, USA), and then dehydrated, embedded in paraffin at 60 °C, sectioned after cooling down (5 μm), stained with hematoxylin-eosin (H&E), and microscopically evaluated. The histomorphometric examination was accomplished using Image J analysis software (National Institutes of Health, USA), including the whole length of mucosa (villus’s tip - the muscular layer), villus’s width (from the villus’s middle), and villus’s height (villus’s tip - crypt junction) [25,26].

### 2.9. Statistical analysis

All results were subjected to one-way ANOVA and expressed as Means ± Standard Error, and then significant variations among groups were determined using Duncan’s multiple range test by SPSS software, version 22. The value of *P* < 0.05 was considered significant.

## 3. Results

### 3.1. Growth performance

Table 2 shows the growth performance results of yellow perch groups incorporated with 2, 4, and 6% Triphala (TR). Triphala-supplemented groups revealed better growth performance compared to the control. The group supplemented with 6% Triphala gave the maximum significant improved average body weight, specific growth rate, body mass gain and length gain rate followed by 4 and 2% incorporated groups compared to the control group (Table 2). Feed conversion ratio and hepatosomatic index showed the best and most significant values (*P* < 0.05) for TR group over the control (Table 2).

**Table 2:** Growth performance indices of yellow perch incorporated with different Triphala levels for four weeks.

Parameters	Triphola %/kg feed			
	0	2	4	6
Initial weight (g)	19.2 ± 1.4	16 ± 1.5	17.1 ± 1.0	19.1 ± 1.9
Final weight (g)	20.5 ± 2.3^d^	28.6 ± 0.5^c^	40.3 ± 2.0^b^	46.7 ± 2.0^a^
Initial length (cm)	12.3 ± 0.1	11.3 ± 0.0	11.3 ± 0.6	12.4 ± 0.3
Final length (cm)	11.5 ± 0.5^d^	13.4 ± 0.3^c^	14.8 ± 0.4^b^	15.2 ± 0.2^a^
SGR (%)	0.2 ± 0.2^d^	0.7 ± 0.0^c^	1.1 ± 0.2^ab^	1.4 ± 0.0^ab^
BMG (%)	13.7 ± 0.6^d^	58.0 ± 0.7^c^	121.7 ± 0.7^b^	158.0 ± 0.8^a^
LGR (%)	13.9 ± 1.0^c^	13.9 ± 1.0^c^	25.5 ± 0.9^ab^	29.4 ± 0.7^ab^
FCR	2.7 ± 0.7^b^	1.7 ± 0.1^a^	0.9 ± 0.2^a^	0.8 ± 0.0^a^
HSI	1.2 ± 0.0^b^	1.8 ± 0.1^a^	2.0 ± 0.1^a^	1.9 ± 0.0^a^

Data are expressed as Mean (n = 90) ± SE and different superscript letters indicate significant difference between groups (*p* < 0.05).

SGR = Specific growth rate, BMG = Body mass gain, LGR = Length gain rate, FCR = Feed conversion ratio and HSI = Hepato somatic index.

### 3.2. Immune response

**Fig 1:**
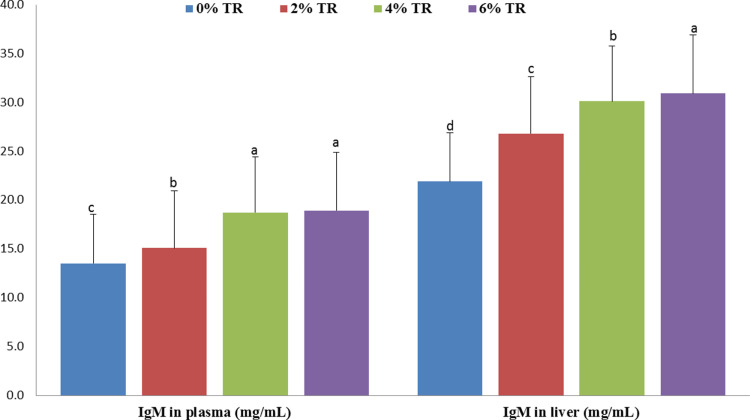
Immunoglobulin M IgM level in plasma and liver of yellow perch receiving 0, 2, 4 and 6% Triphala after four weeks. N = 9 and Means ± SEM with different superscript letters are significantly different (*p* < 0.05).

**Fig 2:**
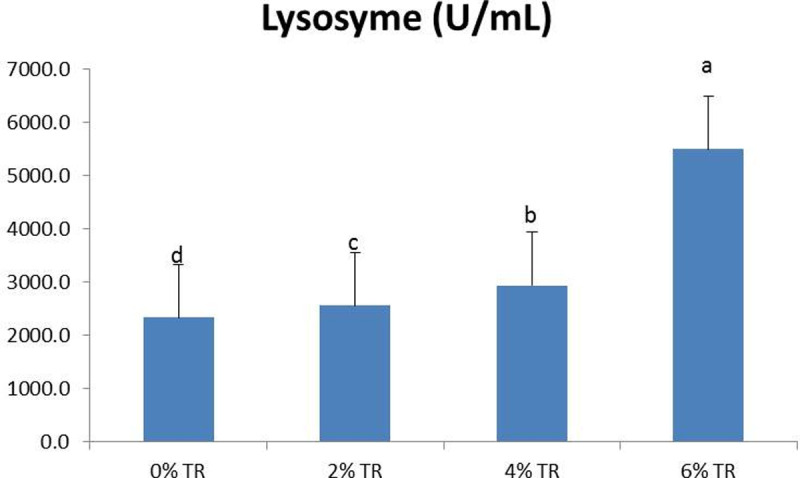
Lysozyme activity in yellow perch after four weeks feeding supplemented diets with 0, 2, 4 and 6% Triphala. Results are described as mean ± SEM (n = 9). Values with different superscript letters are significantly different at (*p* < 0.05).

**Fig 3:**
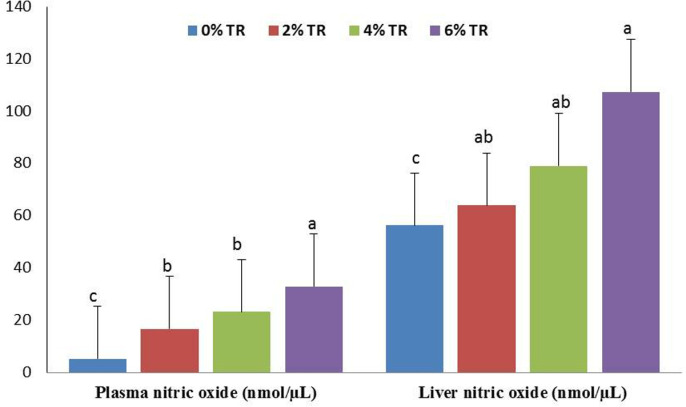
Nitric oxide level in yellow perch plasma and liver incorporated with 0, 2, 4 and 6% Triphala for four weeks. N = 9 and Means ± SEM with different superscript letters are significantly different (*p* < 0.05).

**Fig 4:**
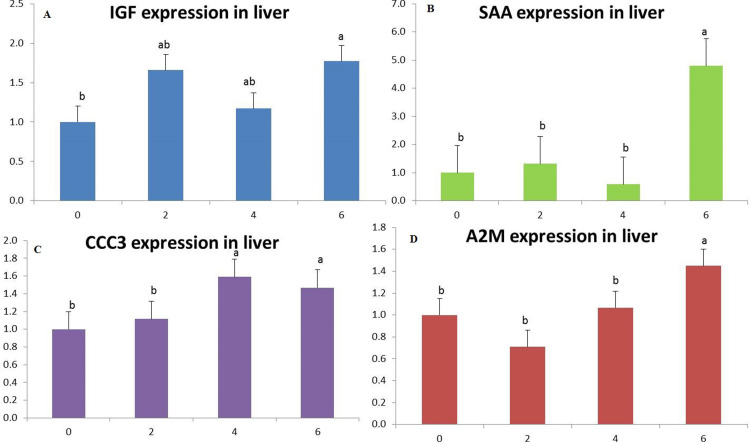
IGF-1 (A), SAA (B), CCC3 (C) and A2M (D) genes expression in yellow perch *P. flavescens* incorporated with 0, 2, 4 and 6% Triphala after four weeks. Results are described as mean ± SEM (n = 9). Values with different superscript letters are significantly different at (*p* < 0.05).

Immune-related parameters are shown in Figs 1–4. Markedly higher IgM levels in both plasma and liver homogenate were recorded in all TR-supplemented groups compared with the control. The most significant increase (*p* < 0.05) was for the 6% group followed by 4% and then 2% (Fig 1), and in plasma both the 6 and 4% group showed the highest result (Fig 1).

Lysozyme activity significantly increased in all TR groups with the 6% group in first place (*p* < 0.05); followed by 4% then 2% over the control group (Fig 2).

NO levels in plasma and liver were significantly higher (*p* < 0.05) in TR groups compared to the control (Fig 3). The 6% TR incorporation gave the most significant (*p* < 0.05) increase followed by 4% and 2% groups, which gave similar significantly higher results (Fig 3).

### 3.2. Glucose and cortisol assays

The TR groups significantly (*p* < 0.05) decreased glucose and cortisol concentrations with the most significant (*p* < 0.05) decrease for the 6% TR group compared to the control (Table 3).

**Table 3:** Effect of different Triphala doses on glucose and cortisol levels of yellow perch.

Parameters	Triphala %/kg feed			
	0	2	4	6
Glucose (g)	1.6 ± 0.0^b^	3.5 ± 0.5^a^	3.8 ± 0.3^a^	2.8 ± 0.2^a^
Cortisol (g)	43.2 ± 0.3^b^	39.7 ± 0.5^ab^	39.3 ± 2.0^ab^	31.4 ± 0.8^a^

Data are expressed as Mean (n = 9) ± SE and different superscript letters indicate significant difference between groups (*p* < 0.05)

### 3.3. Expression of targeted genes

The group supplemented with 6% TR showed the highest (*p* < 0.05) upregulation of IGF-1, SAA, CCC3, and A2M genes expression than the control group (Fig 4 A–D). The group fed with 4% TR diet gave significant upregulation (*p* < 0.05) in both IGF-1 and CCC3 genes expression (Fig 4A and D) and the group supplemented with 2% TR showed significant upregulation (*p* < 0.05) of only IGF-1 gene expression (Fig 4A).

### 3.4. Histological findings

**Fig 5:**
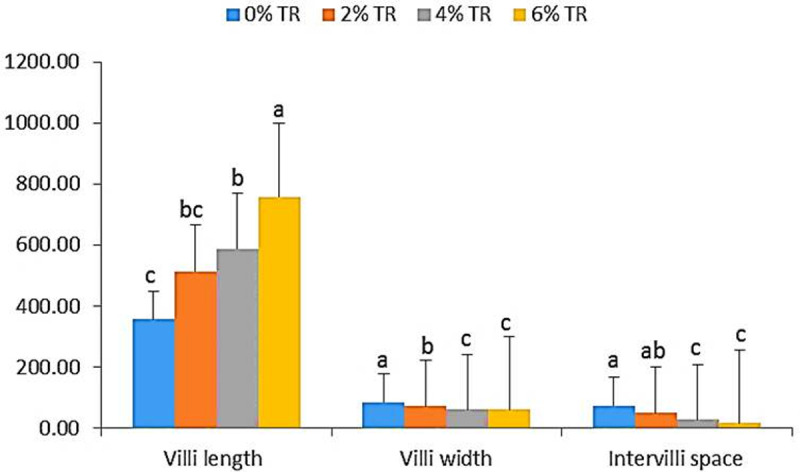
Intestinal morphometry of yellow perch fed diets supplemented with 0, 2, 4 and 6% Triphala. Values are expressed as mean value (n = 9) ± SE. Mean values with different superscript letters are significantly different (*p* < 0.05).

**Fig 6:**
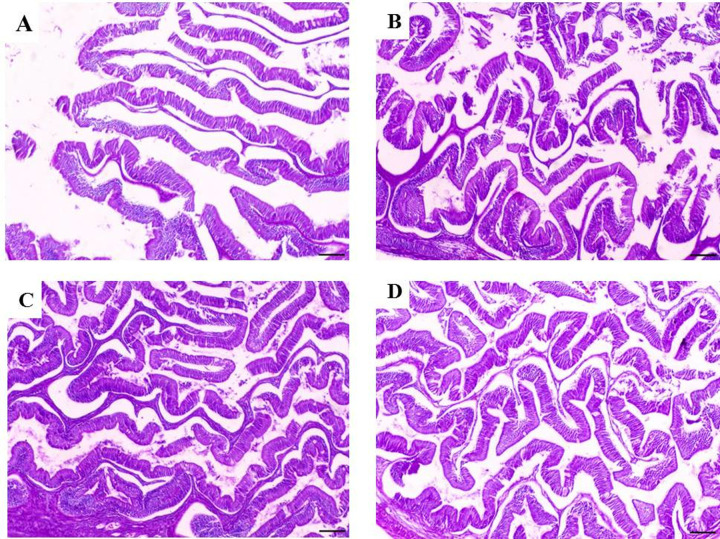
Photomicrograph of the intestine of yellow perch fed diets supplemented with 0, 2, 4 and 6% Triphala after four weeks. A) Middle intestine of yellow perch control group showing villi lined with pseudostratified epithelium, B) Middle intestine of yellow perch supplemented with 2% Triphala showing an increase of intestinal villi length, C) Middle intestine of yellow perch supplemented with 4% of Triphala showing marked increase of intestinal villi length, and D) Middle intestine of yellow perch supplemented with 6% Triphala showing marked increase of intestinal villi length and branches. H&E, X100, bar = 50 µm.

The dietary incorporation with TR showed significant (*p* < 0.05) improvement in intestinal villi length with the most marked increase for the 6% TR group (Figs 5 and 6).

## 3. Discussion

Current study revealed promising growth-promoting activity of Triphala on yellow perch, as it effectively improved the growth performance indices with the best results for the 6% TR group. Triphala is a well-known Indian poly-herbal formula consisting of a combination of three herbal plants *Emblica officinalis*, *Terminalia bellerica*, and *Terminalia chebula* belonging to the *Euphorbiaceae*, *Combretaceae,* and *Combretaceae* families, respectively. Triphala improves growth through the synergistic effect of the active ingredients of its herbal structure, specially its high content of amino acids, fatty acids, carbohydrates [18,19]. This, in turn, can promote good digestion and absorption of food (Baliga et al., 2012; [13,17,27]. Similarly, [16,28] reported maximum significant growth for O*reochromis mossambicus* fed on 0.8% Triphala for 60 days over the control group. In the same instance, *Emblica officinalis* which is one of Triphala’s three herbs, resulted in significantly higher FCR, SGR, condition factor, and weight gain in Rohu (*Labeo rohita*) fingerlings [29]. Current results are also in accordance with the current IGF-1 growth-related gene expression.

In the current study, significantly increased Immunoglobulin M, lysozyme activity, Nitric Oxide, and Myeloperoxidase, specifically in the 6% TR group, conferred the immunostimulating effect of Triphala in yellow perch. Those properties could be linked to the ability of Triphala to mediate immune response, and this is hypothesized to be related to its immunomodulatory and antioxidant properties because of its constituents from potent antioxidants (tannins, gallic acid, and chebulinic acid) [16–18]. Lysozyme, NO, and IgM are essential components for fish’s immune response and their defense capability [30,31]. Triphala showed improvements in the immune response of Nile tilapia [16]. Triphala also showed significant higher lipid peroxidation levels and improved cell-mediated immune response at a concentration of 1% for 48 days in rats [27].

Glucose and cortisol levels are among the biochemical indices that can be used for evaluating fish status and condition [32–34]. In this instance, glucose and cortisol concentrations were markedly decreased in TR-supplemented groups, with the most significant result for 6% TR. This can be due to the ability of Triphala to decrease blood glucose concentration through inhibiting its absorption [17]. This attribution is supported by [35,36], who reported the ability of the most important Triphala components to significantly decrease blood glucose level.

The expression of growth-related gene IGF-I revealed upregulation for the TR groups over the control group with the most noticeable upregulation for the 6% TR group, and this supports our current growth performance findings. This is related to the growth-promoting properties of Triphala and its ability to improve the feed uptake, utilization, and absorption through its active ingredients of amino acids, fatty acids, and carbohydrates [18,19]. In addition, the expression profiles of immune-related genes SAA, CCC3, and A2M showed the maximum expression for the 6% TR-incorporated group, which are also in agreement with our current results on immune response. This upregulation is also due to the active components of *the positive synergestic activity between the herbal content of Triphalaa,* which are well known to stimulate immune and anti-oxidative responses [17,18]. Additionally, it has been reported that Triphala effectively mitigated alterations in lipid peroxidation at concentration of 1 g for 48 days in rats [37]. Moreover, Triphala at a dose of 3 mg increased the lysozyme activity in treated mice [38].

Intestinal histology reading is correlated with growth performance and immune response parameters data, revealing the most improved findings for the group enriched with 6% TR. This revealed a remarked development of the intestinal villi length that continued to progress over time throughout the entire experimental period compared to the control group. This finding may be due to the ability triphala to sustain the intestinal contour and restore exhausted protein concentrations on the brush border of villi; and this was obvious in data reported by [16] who found significantly improved intestinal villi length in Nile tilapia incorporated with triphala compared to the control group. Similarly, [12] reported improved intestinal histology in groups supplemented with curcumin and chitosan. In addition, other herbal natural supplementations were proved to significantly enhance the intestinal morphometry of *Oreochromis mossambicus* and *Clarias gariepinus* [39]. These results are consistent with the growth and antioxidants readings, which confirm the triphala positive properties.

With the arising issue of antimicrobial resistance that results from chemical and antibiotic usage, the need for safe, natural alternatives becomes a must. Accordingly, our current findings from the yellow perch study concluded that Triphala gave promising results for growth, immune response, related gene expression, and gut morphometry. With the lack of previous studies of the effect of Triphala on yellow perch and its gut morphometry and the effective lowering of cortisol level results, more investigations addressing its antioxidant activity are required.
